# Correlation between antibacterial activities of two *Artemisia* spp. extracts and their plant characteristics

**DOI:** 10.14440/jbm.2024.0116

**Published:** 2025-06-19

**Authors:** Abdullah Mashraqi, Mohamed A. Al Abboud, Khatib Sayeed Ismail, Yosra Modafer, Mukul Sharma, A. El-Shabasy

**Affiliations:** 1 Department of Biology, College of Science, Jazan University, Jazan 45142, Kingdom of Saudi Arabia; 2 Environment and Nature Research, Jazan University, Jazan 45142, Kingdom of Saudi Arabia

**Keywords:** *Listeria*, *Pseudomonas*, *Staphylococcus*, Morphology, Correlation, Palynology, Anatomy, Adaptation

## Abstract

**Background:**

The antimicrobial activity of *Artemisia absinthium* L. and *Artemisia herba-alba* Asso. against various pathogens is differentiated by using different organic solvents and aqueous solution and in terms of pollen grain traits. The similarities and dissimilarities were analyzed by simple linear regressions and in terms of Pearson correlation coefficients.

**Objective:**

The present study evaluated the potential antibacterial activity of *A. absinthium* L. and *A. herba-alba* Asso. extracts by using various organic and aqueous solvents. The tested bacteria included pathogenic strains: *Listeria monocytogenes, Pseudomonas aeruginosa, Salmonella enterica*, and *Staphylococcus aureus*.

**Methods:**

Different affinities were observed for the studied organic solvents in addition to aqueous ones. A comparative analysis was conducted, focusing morphological, anatomical, and palynological characteristics. The similarity parameter was obtained. The minimum inhibitory concentration (MIC) values for both plant extracts were analyzed using the analysis of variance, while Pearson correlation coefficients were calculated for plant traits.

**Results:**

Butanol emerged as the predominant organic solvent extract for both species whereas chloroform and diethyl ether exhibited a broad antibacterial spectrum for *A. absinthium* L. and *A. herba alba* Asso. MIC and minimum bactericidal concentration values were confirmed by using butanol and diethyl ether extracts of *A. absinthium* L. and butanol and chloroform extracts of *A. herba alba* Asso. against the tested pathogenic bacteria. The results highlight the potential of these extracts as alternative natural antibacterial agents.

**Conclusion:**

This study demonstrated that using successive organic plant extractions can help identify the most effective extract that can serve as a source of alternative medicine due to its various active natural components.

## 1. Introduction

The majority of phytochemical secondary metabolites are biosynthetically derived from primary metabolites in plants. These compounds can be categorized into several groups based on their medical value. Among the active and therapeutic phytochemicals effective in treating a wide range of illnesses are alkaloids, tannins, steroids, volatile oils, glycosides, fixed oils, phenols, resins, and flavonoids. The phytochemicals are predominantly found in various plant parts, including flowers, leaves, seeds, bark, roots, and fruits.^1^,^2^

Numerous medicinal plants have demonstrated antimicrobial activities against a wide range of infectious and harmful bacteria. Due to their environmental safety and lack of adverse effects, researchers increasingly favor natural alternatives over synthetic additives. As a result, published research focuses on employing plant extracts as natural antibiotics in accordance with the recommendations from healthcare professionals.^3^,^4^

The *Artemisia* genus within the *Asteraceae* family holds the significant economic value. This genus is known for its diverse medicinal benefits, including antimicrobial, anticancer, antirheumatic, and antispasmodic properties. While some species can be toxic or allergenic, many are used as medicinal plants.^5^,^6^ The *Artemisia* genus comprises approximately 500 species distributed across the northern hemisphere, including the regions in Asia, America, and Europe. Notably, *Artemisia absinthium* L. and *Artemisia herba-alba* Asso. are two prominent medicinal herbs that exhibit extensive geographical adaptability across various habitats, mainly in arid and semi-arid regions. This adaptability stimulates the production of diverse phytochemical constituents tailored to their environments.^7^

The infrageneric taxonomy of this economically significant genus remains a complex and ambiguous endeavor due to their intricate nature of plant structures. The morphology, anatomy, pollen grain, and cypselas of *Artemisia* and its allied species present considerable challenges for plant taxonomists. This difficulty in differentiation may arise from the diverse ecological conditions in which *Artemisia* species thrive, particularly in arid and semi-arid regions where they exhibit a wide range of geographical adaptations.^8^

The objective of this study was to examine the antibacterial properties of extracts from *A. absinthium* and *A. herba-alba* using various organic and aqueous solvents against selected pathogenic bacteria. These plant species originate from different geographical regions. The antibacterial activity observed in these species correlates with the findings of other plant taxonomical studies, including morphological, anatomical and palynological analyses.

## 2. Materials and methods

### 2.1. Plant collection and identification

**Figure 1:**
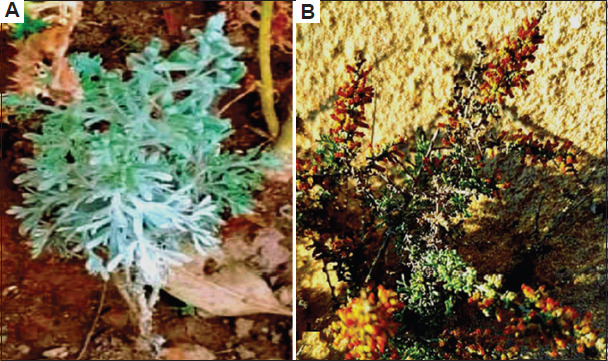
The studied plant species. (A) *Artemisia absinthium* L. and (B) *Artemisia herba-alba* Asso.

**Figure 2:**
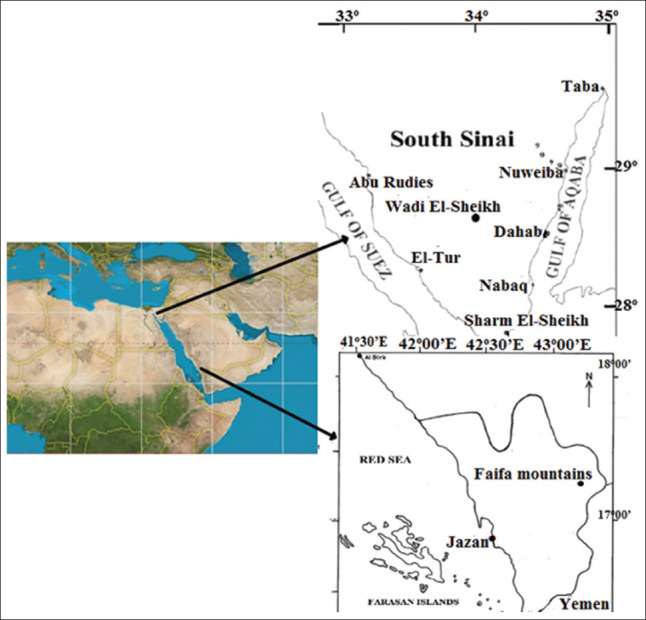
The area of plant collection; (A): *Artemisia herba-alba*, (B): *Artemisia absinthium*

The plant material *A. absinthium* was collected from the Faifa mountains in Jazan Saudi Arabia, located at a latitude of 17°15′55.3′′ and a longitude of 43°06′47.1′′, at an elevation of 383 m in September 2022. Conversely, *A. herba-alba* was harvested from the Wadi El-Sheikh area in Sinai, Egypt, situated at a latitude of 28°50′15.3′′and a longitude of 33°55′30.1′′, at an elevation of 77 in July 2022 (Figures 1 and 2). The herbarium of the Biology Department, College of Science, Jazan University, confirmed the identification of the studied plant species. The entire plant specimens were dried in a hot air oven at 50°C for 24 h and subsequently ground into powder. This powdered plant material was used for extractions with various aqueous and other organic solvents: acetone, butanol, chloroform, diethyl ether, ethanol, and methanol. Each whole plant extract was prepared by following the method described previously.^9^ Specifically, 20 g of each plant material was placed in 250 mL of the studied respective solvent and stirred using a water bath at 45°C for 10 h. After that, the plant residue was separated by filtration through a Whatmann No. 1 filter paper and the solvent was evaporated under vacuum on a rotary evaporator at 40°C (Rotavapor R-200, Büchi Labortechnik, Switzerland) except for the aqueous solvent. The resulting residue was dissolved in 50 mL of dimethyl sulfoxide (DMSO) and stored at −20°C for further use. This final step was not performed during the aqueous solvent preparation. Three replicates were conducted for each solvent extraction, and standard deviations (SD) were obtained.^10^

Plant materials were collected from (A) the Wadi El-Sheikh area in Sinai, Egypt,^11^ and (B) the Faifa Mountains in Jazan, Saudi Arabia.^12^

### 2.2. Bacterial strains

The antibacterial potency of each plant extract was assessed against four pathogenic bacterial strains, which were isolated, identified and accredited by the American Type Culture Collection (ATTC) international accredited company.

ATTC was established in 1925 when a committee of scientists recognized the need for a central repository of microorganisms that scientists worldwide could use to advance the field of microbiology. ATCC has established catalogs featuring more than 2,000 strains, complete with extensive cross-referencing information and trademarks. All the summarized data of studied bacterial strains are presented in Tables 1 and 2.

**Table 1:** Clinical and microbiological characteristics of studied bacterial strains as provided by the American Type Culture Collection

Property		*Pseudomonas aeruginosa*	*Salmonella enterica*	*Staphylococcus aureus*
Specific applications	Media testing, enteric research, food testing	Opportunistic pathogen research	Control culture, media testing, preparatory test control, enteric research, emerging infectious disease research, pharmaceutical and personal care, and water testing	Christie-Atkinson-Munch-Peterson test, assay of wood, smoke concentrate, control strain, control strain for identification, evaluation of Mueller-Hinton agar, examination of dairy products, media testing quality control strain, reference material, susceptibility disc testing, enteric research, and water testing
Temperature	37°C	37°C	37°C	37°C
Atmosphere	Aerobic	Aerobic	Aerobic	Aerobic
Isolation source	Poultry	Clinical isolate from human	Tissue from pools of heart and liver from 4-week-old chickens	Clinical isolate from human
Applications	Enteric disease research, food testing, infectious disease research, media testing, and zoonotic disease research	Enteric disease research, food testing, infectious disease research, media testing, and zoonotic disease research	Bioinformatics enteric disease research food testing infectious disease research media testing water testing quality control, and pharmaceutical testing	Bioinformatics, enteric disease research, infectious disease research, media testing, quality control, and water testing
Product format	Freeze-dried	Freeze-dried	Freeze-dried	Freeze-dried
Storage conditions	2°C – 8°C	2°C – 8°C	2°C – 8°C	2°C – 8°C
Susceptibility profile	-	Gentamicin	-	Ampicillin, caryomycin, cephalexin, and cephaloglycin

Note: Information in the table was adopted from Chaudhary *et al*^13^.

**Table 2:** Genomic data of selected bacterial strains as provided by the American Type Culture Collection

Genomic subunits		*Pseudomonas aeruginosa*	*Salmonella enterica*	*Staphylococcus aureus*
Number of coding DNA sequence	2,845	6,379	4,644	2,576
Number of hypothetical proteins	1,025	2,762	1,253	928
Number of translational RNA	53	4	86	60
Number of 5s ribosomal RNA	1	4	8	7
Number of 16s ribosomal RNA	1	4	7	6
Number of 23s ribosomal RNA	1	4	7	6

Note: Information in the table was adopted from Chaudhary *et al*^13^.

*Listeria monocytogenes*^14^ (Pirie, ATCC^®^ 19111™) is a mesophilic bacterium belonging to *Bacillota* phylum, *Bacilli* class, *Caryophanales* order, and *Listeriaceae* family. It is the causative agent of listeriosis, a Gram-positive, facultatively intracellular rod capable of causing reproductive disease, neurological disease, and septicemia in a wide range of hosts. It is regarded as a highly consequential human foodborne pathogen because of its ubiquitous presence in the environment and ability to cause devastating diseases in veterinary species, especially ruminants. *L. monocytogenes* tolerates a wide range of temperatures, capable of replicating when refrigerated at 5°C and surviving in some pasteurization techniques, as well as tolerating a broad pH range.^15^

*Pseudomonas aeruginosa* (Schroeter) Migula (NCTC 10662/ATCC^®^ 25668™) is an aerobic Gram-negative bacillus, and an opportunistic pathogen. It can tolerate low-oxygen conditions due to its highly versatile nature, grow in temperature ranging from 4°C to 42°C and survive with low levels of nutrients. On medical equipment and in hospital environments, where it infects immunocompromised patients, causing bacteremias, urinary tract infections, and pneumonias. It causes high mortality and morbidity rates in patients with cystic fibrosis, eventually leading to respiratory insufficiency and pulmonary damage.^16^

*Salmonella enterica* subspp. *enterica* (exKauffmann and Edwards) Le Minor and Popoff serovar Typhimurium (ATCC^®^ 14028™) falls into the Gamma proteobacteria class and belongs to the *Enterobacteriaceae* family. It is a Gram-negative, rod-shaped, and non-spore-forming bacterium that is facultatively anaerobes and primarily exhibits peritrichous motility.^17^ Propulsion is achieved via three or four flagella that arise randomly from its sides and extend into the external medium.^18^

*Staphylococcus aureus* subspp. *aureus* Rosenbach strain Seattle 1945 (ATCC® 25923™) belongs to *Bacillota* phylum, *Bacilli* class, *Bacillales* order, and *Staphylococcaceae* family. It is a Gram-positive bacteria, non-spore-forming, facultatively anaerobic and non-motile bacteria. *S. aureus* can grow through fermentation or aerobic respiration, resist heat and tolerate high concentrations of salts. It is a highly virulent opportunistic pathogen that causes a variety of infectious diseases and food poisoning.^19^,^20^

### 2.3. Evaluation of the antibacterial activity of plant extracts

The disc-diffusion assay was used to assess the antibacterial activity of the plant extracts. The media composition for each bacteria strain is detailed in Table 3. The steps for culture preparation were conducted according to the standard procedures. First, the vial was opened following the enclosed instructions. Using a single tube of #44 broth (5 – 6 mL), approximately 0.5 – 1.0 mL was withdrawn with a Pasteur or a 1.0 mL pipette, and the entire pellet was rehydrated. This aliquot was aseptically transferred back into the broth tube and mixed thoroughly. Several drops of the suspension were used to inoculate agar slants and plates. Finally, the tubes and plate were incubated at 37°C for 24 h.^21^ Each plant residue, dissolved in 50 mL of DMSO, was dripped onto sterile filter paper discs (9 mm in diameter, Whatmann No. 3 chromatographic paper). Each filter paper disc was loaded with 2 mg of the respective plant extract. These discs were subsequently placed on agar medium plates inoculated with reference bacterial strains from the ATCC used for antimicrobial screening and incubated at 35°C ± 2.5°C for 24 h. A paper disc containing DMSO was used as a negative control. Commercial 6-mm diameter discs containing 0.01 mg of streptomycin were employed as a positive control. The diameter of the clear zone surrounding the plant extract-loaded discs was measured in millimeters using a Vernier caliper to evaluate the antibacterial activity of the extracts.^4^

**Table 3:** Media accredited by the American Type Culture Collection for studied bacterial strains

American Type Culture Collection Medium		*Pseudomonas aeruginosa*	*Salmonella enterica*	*Staphylococcus aureus*
Medium Name	44 Brain Heart Infusion Agar/Broth	Nutrient agar/broth No. 3	Nutrient agar/broth No. 3	18 Tryptic soy agar/broth (Soybean-casein digest medium, USP)
Agar medium composition	52 g Brain Heart Infusion agar (BD 211065), and 1000 mL DI water	23 g Nutrient agar (BD 213000), and 1000 mL DI water	23 g Nutrient agar (BD 213000), and 1000 mL DI water	40 g Tryptic soy agar (BD 236950), and 1000 mL DI water
Broth medium composition	37 g Brain–Heart Infusion broth (BD 237500), and 1000 mL DI water	8 g Nutrient broth (BD cat 234000), and 1000 mL DI water	8 g Nutrient broth (BD cat 234000), and 1000 mL DI water	30 g Tryptic soy broth (BD cat 211825), and 1000 mL DI water
Brain Heart Infusion Composition	200 g infusion from calf brains, 250 g infusion from beef hearts, 10 g protease peptone, 2 g dextrose, 5 g NaCl, 2.5 g Na_2_HPO_4_, and 1000 mL DI water	Nil	Nil	Nil
Nutrient agar composition	Nil	3 g beef extract, 5 g peptone, and 15 g agar	3 g beef extract, 5 g peptone, and 15 g agar	Nil
Tryptic soy agar composition	Nil	Nil	Nil	17 g tryptone, 3 g soytone, 2.5 g dextrose, 5 g NaCl, 2.5 g K_2_HPO_4_, and 15 g agar

Note: Information in the table was adopted from Na-Bangchang *et al*^13^. Abbreviations: BD: Becton Dickinson; cat: Catalogue DI: Deionized; USP: United States Pharmacopeia.

### 2.4. Determination of the minimum inhibitory concentration (MIC) of plant extracts

MIC represents the lowest antimicrobial concentration that inhibits microbial growth after 24 h of incubation. For each tested bacterium, the MIC was determined through a serial micro-dilution of plant extract in DMSO solution with concentrations ranging from 6.36 to 392.0 mg/L, by following the previously described protocol.^22^ The inhibition zones were measured by a Vernier caliper and documented in relation to the concentrations of the effective plant extracts.

### 2.5. Assessment of the minimum bactericidal concentration (MBC)

MBC represents the lowest plant extract concentration which shows no bacterial growth following the MIC assessment. Freshly inoculated agar plates were incubated at 37°C for 24 h. For each plate, three separate biological replicates were conducted, and the absence of colonies on the plates indicated effective bactericidal activity.^23^

### 2.6. Plant characterizations

Plant characterizations were conducted to identify both qualitative and quantitative traits. Qualitative traits describe plant parts while quantitative traits are measurable characteristics. Morphological traits were categorized into seven groups: habitat, leaf, stem, petiole, bract, inflorescence, and cypselas. Each category included numerous subcharacteristics denoted with (+) for presence or (-) for absence. Moreover, anatomical traits were classified into five groups: leaf, stem, root, leaf epidermis, and stomata. All anatomical observations and measurements were performed according to the established protocols.^6^,^24^,^25^ Cypsela features were assessed by the following established guidlines.^26^ The symbol (+) indicates presence, while (-) indicates absence. Scanning electron microscopic images of pollen grains were captured based on a previously described method.^27^ The measurements of the polar axis, equatorial axis, polar axis/equatorial axis, sphericity, exine thickness, and aperture length were taken according to the previous protocol.^27^,^28^. All other palynological characteristics were measured from the previous photos as new investigations. These characteristics were also classified also into qualitative and quantitative traits. A glossary of all abbreviations used is presented in the Appendix.

### 2.7. Statistical analysis

Pearson correlation coefficients among the MIC values of *A. absinthium* and *A. herba alba* extracts against the tested bacterial strains were calculated according to a previous method.^29^ *P* values from the significance tests based on the degrees of freedom were determined according to a previously used approach.^30^ The MIC values for each plant extract were subjected to the statistical analysis using the analysis of variance (ANOVA) to compare means and standard error (SE) was calculated using a previously described method.^31^

Statistical tests were conducted using the Statistical Package for the Social Sciences software (version 22) for Windows.^12^ The significant relationships were further analyzed and represented using simple linear regression (SLR) based on previous methods. This was performed for each bacterial strain individually against two studied plant extracts and for comparative data between the two plant species. They were done using linear regression approaches which explored the extent of the effect of comparative parameters on observed relationships.^32^,^33^

## 3. Results

### 3.1. Analysis of antibacterial activity

**Figure 3:**
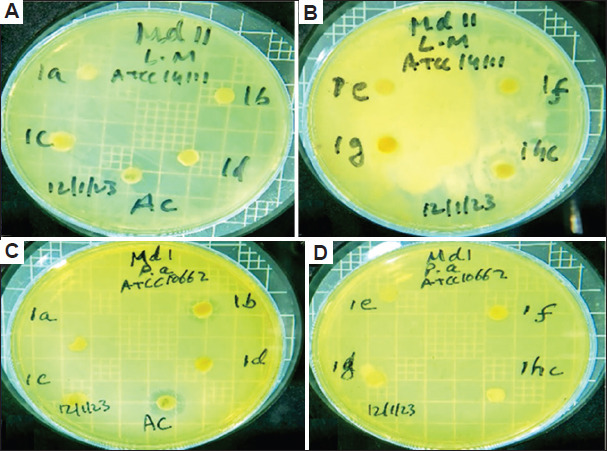
Diameter of inhibition zone of *Artemisia absinthium*. The inhibition zone of *A. absinthium* against (A and B) *Listeria monocytogenes* (L m) and (C and D) *Pseudomonas aeruginosa* (P a) when different extract solvents were used. Abbreviations: a: Acetone; AC: Streptomycin; b: Butanol; c: Chloroform; d: Diethyl ether; E: Ethanol; F: Aqueous solution; hc: Dimethylsulfoxide.

**Figure 4:**
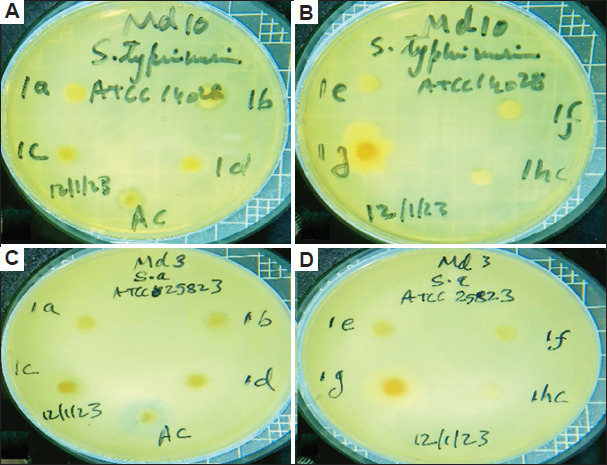
Diameter of inhibition zone of *Artemisia absinthium*. The inhibition zone of *A. absinthium* against (A and B) *Salmonella enterica* (S e) and (C and D) *Staphylococcus aureus* (S a) when different extract solvents were used. Abbreviations: a: Acetone; AC: Streptomycin; b: Butanol; c: Chloroform; d: Diethyl ether; E: Ethanol; F: Aqueous solution; hc: Dimethylsulfoxide.

**Figure 5:**
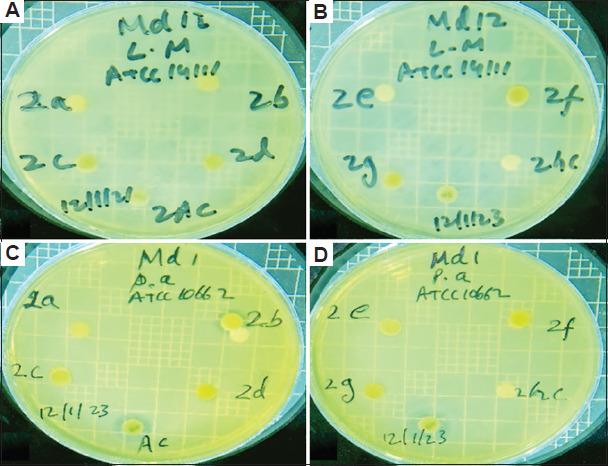
Diameter of inhibition zone of *Artemisia herba-alba*. The inhibition zone of *A. herba-alba* against (A and B) *Listeria monocytogenes* (L m) and (C and D) *Pseudomonas aeruginosa* (P a) when different extract solvents were used. Abbreviations: a: Acetone; AC: Streptomycin; b: Butanol; c: Chloroform; d: Diethyl ether; E: Ethanol; F: Aqueous solution; hc: Dimethylsulfoxide.

**Figure 6:**
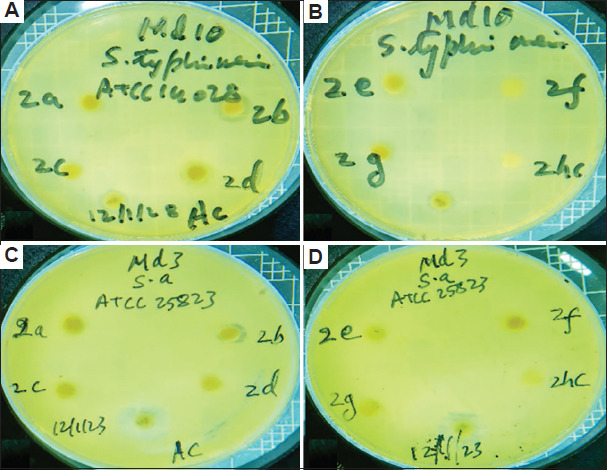
Diameter of inhibition zone of *Artemisia herba-alba*. The inhibition zone of *Artemisia herba-alba* against (A and B) *Salmonella enterica* (S e) and (C and D) *Staphylococcus aureus* (S a) using different extract solvents. Abbreviations: a: Acetone; AC: Streptomycin; b: Butanol; c: Chloroform; d: Diethyl ether; E: Ethanol; F: Aqueous solution; hc: Dimethylsulfoxide.

**Figure 7:**
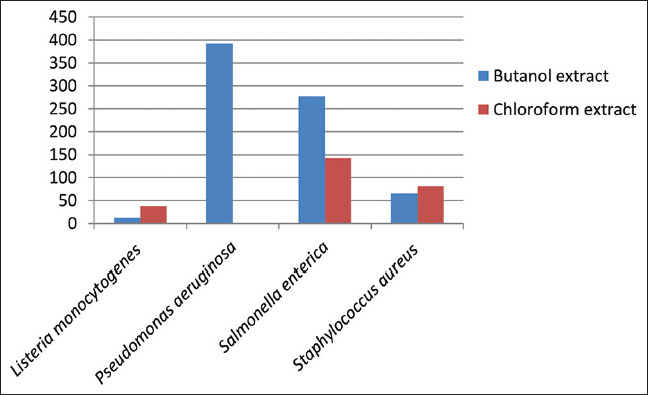
The minimum inhibitory concentration of *Artemisia absinthium* extracts against the tested bacterial strains

**Figure 8:**
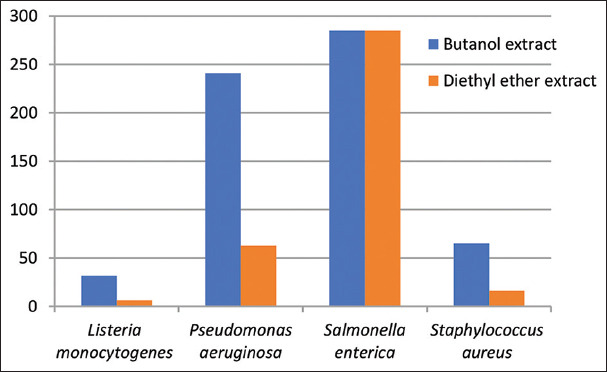
The minimum inhibitory concentration of *Artemisia herba-alba* extracts against the tested bacterial strains

The antibacterial activities of different solvent extracts of the two studied plant species in terms of the diameters of the inhibition zones (IZ), MIC, and MBC are presented in Tables 4-9 and Figures 3-8.

**Table 4:** Antibacterial activity of *Artemisia absinthium* extracts against the tested bacterial strains

Bacterial strain	Diameter of inhibition zone (mm)								

	Organic solvents						Aqueous solvent	DMSO	Streptomycin

	Acetone	Butanol	Chloroform	Diethyl ether	Ethanol	Methanol			
	-	1.0±0.01	3.0±0.01	-	-	2.5±0.01	-	-	12.0±0.01
*Pseudomonas aeruginosa*	-	5.5±0.58	-	-	-	-	-2.75±1.71	-	7.0±0.01
*Salmonella enterica*	-	3.88±0.25	2.0±0.01	-	-	-	-11.0±4.08	-	9.5±0.01
*Staphylococcus aureus*	-	2.0±0.01	2.5±0.01	-	-	-	-3.5±0.01	-	11.0±0.01

Abbreviation: DMSO: Dimethyl sulfoxide.

**Table 5:** Antibacterial activity of *Artemisia herba-alba* extracts against the tested bacterial strains

Bacterial strain	Diameter of inhibition zone (mm)								

	Organic solvents						Aqueous solvent	DMSO	Streptomycin

	Acetone	Butanol	Chloroform	Diethyl ether	Ethanol	Methanol			
	-	2.5±0.01	-	0.5±0.01	-	-	-	-	9.0±0.01
*Pseudomonas aeruginosa*	-	3.38±0.48	-	0.88±0.25	-	-	-	-	8.0±0.01
*Salmonella enterica*	-	4.0±0.01	-	4.0±0.01	-	-	-	-	3.5±0.01
*Staphylococcus aureus*	-	2.0±0.01	-	0.5±0.01	-	-	-	-	8.0±0.01

Abbreviation: DMSO: Dimethyl sulfoxide.

**Table 6:** MICs of *Artemisia absinthium* extracts against the tested bacterial strains

Bacterial strain	MIC (mg/mL)							

	Organic solvents						Aqueous solvent	DMSO

	Acetone	Butanol	Chloroform	Diethyl ether	Ethanol	Methanol		
	-	12.71±0.01	38.14±0.01	-	-	31.78±0.01	-	-
*Pseudomonas aeruginosa*	-	392.0±0.58	-	-	-	-	-	-
*Salmonella enterica*	-	276.55±0.25	142.55±0.01	-	-	-	-	-
*Staphylococcus aureus*	-	65.14±0.01	81.43±0.01	-	-	-	-	-
*F* test	Lm vs Pa 51.01	Lm vs Se 31.10	Lm vs Sa 3.44	Pa vs Se 1.64		Pa vs Sa 14.82		Se vs Sa 9.03
(Analysis of variance)	***	***	*	*		***		***

Note: Statistical significance determined at *p<*0.05 *, *p<*0.01**, and *p<*0.001***. Abbreviations: DMSO: Dimethyl sulfoxide; Lm: *Listeria monocytogenes*; Pa: *Pseudomonas aeruginosa*; Sa: *Staphylococcus aureus*; Se: *Salmonella enterica*; vs: Versus; MIC: Minimum inhibitory concentration.

**Table 7:** MICs of *Artemisia herba* alba extracts against the tested bacterial strains

Bacterial strain	MIC (mg/mL)							

	Organic solvents						Aqueous solvent	DMSO

	Acetone	Butanol	Chloroform	Diethyl ether	Ethanol	Methanol		
	-	31.78±0.01	-	6.36±0.01	-	-	-	-
*Pseudomonas aeruginosa*	-	240.91±0.48	-	62.72±0.25	-	-	-	-
*Salmonella enterica*	-	285.10±0.01	-	285.10±0.01	-	-	-	-
*Staphylococcus aureus*	-	65.14±0.01	-	16.28±0.01	-	-	-	-
*F* test	Lm vs Pa 43.49	Lm vs Se 111.61	Lm vs Sa 3.16	Pa vs Se 2.57		Pa vs Sa 13.76		Se vs Sa 35.30
Analysis of variance	***	***	**	*		***		***

Note: Statistical significance determined at *p<*0.05 *, *p<*0.01**, and *p<*0.001***. Abbreviations: DMSO: Dimethyl sulfoxide; Lm: *Listeria monocytogenes*; Pa: *Pseudomonas aeruginosa*; Sa: *Staphylococcus aureus*; Se: *Salmonella enterica*; vs: Versus; MIC: Minimum inhibitory concentration.

**Table 8:** MBCs of the tested bacterial strains against *Artemisia absinthium* extracts

Bacterial strain	MBC (mg/mL)							

	Organic solvents						Aqueous solvent	DMSO

	Acetone	Butanol	Chloroform	Diethyl ether	Ethanol	Methanol		
	-	8.0±0.01	24.01±0.01	-	-	20.0±0.01	-	-
*Pseudomonas aeruginosa*	-	2.0±0.58	-	-	-	-	-	-
*Salmonella enterica*	-	2.0±0.25	1.03±0.01	-	-	-	-	-
*Staphylococcus aureus*	-	4±0.01	5±0.01	-	-	-	-	-

Abbreviations: DMSO: Dimethyl sulfoxide; MBC: Minimum bacterial concentration.

**Table 9:** MBC of the tested bacterial strains against *Artemisia herba-alba* extracts

Bacterial strain	MBC (mg/mL)							

	Organic solvents						Aqueous solvent	DMSO

	Acetone	Butanol	Chloroform	Diethyl ether	Ethanol	Methanol		
	-	20.0±0.01	-	4.0±0.01	-	-	-	-
*Pseudomonas aeruginosa*	-	1.23±0.48	-	0.32±0.25	-	-	-	-
*Salmonella enterica*	-	2.06±0.01	-	2.06±0.01	-	-	-	-
*Staphylococcus aureus*	-	4.0±0.01	-	1.0±0.01	-	-	-	-

Abbreviations: DMSO: Dimethyl sulfoxide; MBC: Minimum bacterial concentration.

The butanol extract of *A. absinthium* revealed antibacterial effects against all the tested bacterial stains. The highest antibacterial activity was observed against *P. aeruginosa* (IZ 5.5 mm) while the lowest activity was recorded against *L. monocytogenes* (IZ 1.0 ± 0.01). Conversely, chloroform extract exhibited similar effects except on *P. aeruginosa*. The highest antibacterial activity was exhibited against *L. monocytogenes* (IZ 3.0 ± 0.01 mm) while the lowest activity was observed against *S. enterica* (IZ 2.0 ± 0.01 mm). In contrast, the methanol extract displayed antibacterial activity solely on *L. monocytogenes* (IZ 2.5 ± 0.01 mm). Butanol and diethyl ether extracts of *A. herba alba* showed antibacterial activity on all tested pathogenic bacteria. The highest antibacterial activity was observed against *S. enterica* in both plant extracts, with an IZ value of 4.0 ± 0.01 mm. At the same time, the lowest activity was observed against *S. aureus*, with an IZ value of 2.0 ± 0.01 and 0.5 ± 0.01 mm in butanol and diethyl ether extracts, respectively. Moreover, *L. monocytogenes* presented a comparable antibacterial activity against *S. aureus* when the diethyl ether extract was used.

Despite aqueous solvents showing no effects on bacterial growth using *A. herba alba* extracts, there was an increase in growth when *A. absinthium* was used against all tested pathogenic bacteria except *L. monocytogenes*. DMSO exhibited no inhibitory activity. Streptomycin exhibited significant antibacterial activity against all tested bacteria.

With regard to the MIC values, *L. monocytogenes* showed the lowest concentration (12.71 ± 0.01 mg/mL) while *P. aeruginosa* exhibited the highest concentration (392.0 ± 0.58 mg/mL) when butanol extracts of *A. absinthium* were used. In contrast, for chloroform treatment, *L. monocytogenes* had the highest concentration (38.14 ± 0.01 mg/mL) while *S. aureus* yielded the lowest concentration (81.43 ± 0.01 mg/mL). The methanol extract presented an MIC value of 31.78 ± 0.01 mg/mL against *L. monocytogenes*. For *A. herba alba* extracts, *S. enterica* also demonstrated the highest MIC value of 285.10 ± 0.01 mg/mL in both organic extracts. In contrast, *L. monocytogenes* showed the lowest MIC values of 31.78 ± 0.01 mg/mL and 6.36 ± 0.01 mg/mL in butanol and diethyl ether extracts, respectively.

The MBC values ranged from 1.03 ± 0.01 to 8.0 ± 0.01 mg/mL in butanol and chloroform extracts of *A. absinthium*, but *L. monocytogenes* exhibited an MBC value of 24.01 ± 0.01 mg/mL in the chloroform extract. In contrast, the MBC values for *A. herba alba* extracts ranged from 0.32 ± 0.25 to 4.0 ± 0.01 mg/mL with *L. monocytogenes* showing an MBC value of 20.0 ± 0.01 mg/mL in the butanol extract.

### 3.2. Analysis of plant characteristics

**Figure 9:**
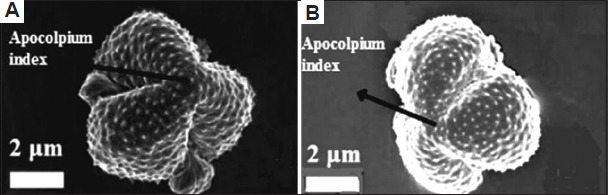
Scanning electron microscopic images of pollen grains. Apocolpium indexes of pollen grains for (A) *Artemisia absinthium* and (B) *Artemisia herba-alba*. Scale bar: 2 µm.

**Figure 10:**
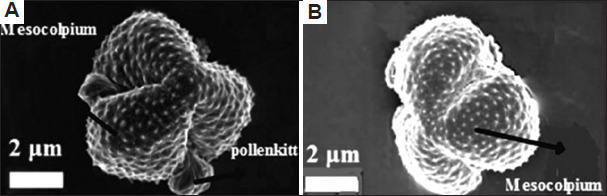
Scanning electron microscopic images of pollen grains. Mesocolpia of pollen grains for (A) *Artemisia absinthium* and (B) *Artemisia herba-alba*. Scale bar: 2 µm.

**Figure 11:**
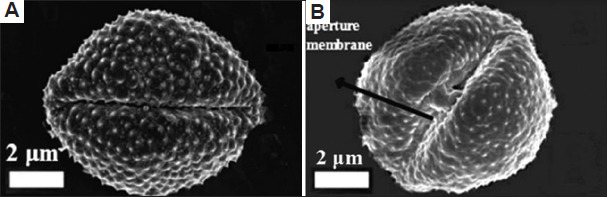
Scanning electron microscopic images of pollen grains. Apertures of pollen grains for (A) *Artemisia absinthium* and (B) *Artemisia herba-alba*. Scale bar: 2 µm.

**Figure 12:**
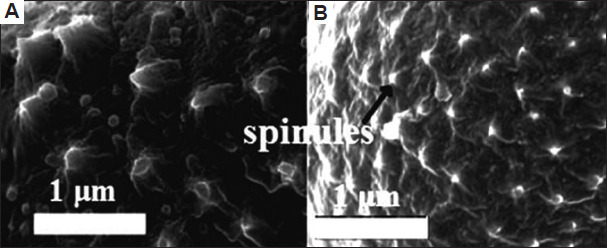
Scanning electron microscopic images of pollen grains. Spinules of pollen grains for (A) *Artemisia absinthium* and (B) *Artemisia herba-alba*. Scale bar: 1 µm.

A total of 43 morphological traits were documented from both studied plant species, classified into 34 qualitative and nine quantitative traits across seven groups: Habitat (seven traits), leaf (10 traits), stem (four traits), petiole (one trait), bract (four traits), inflorescence (13 traits), cypsela (four traits) (Table 10). Similarly, 66 anatomical characteristics were recorded for both plant species (48 qualitative and 18 quantitative) distributed across five groups: leaf (13 traits), stem (17 traits), root (eight traits), leaf epidermis (eight traits), stomata (20 traits) (Tables 11 and 12). Moreover, 25 palynological traits were analyzed, consisting of 12 qualitative and 13 quantitative ones. Each plant trait was symbolized as either (+) for presence, (-) for absence (Table 13 and Figures 9-12). The similarity percentage was measured for each plant group’s characteristics, as shown in Table 14.

**Table 10:** Morphological characteristics of the two plant species studied

Morphological characters	*Artemisia absinthium*	*Artemisia herba-alba*
Habit	Perennial (+)	Perennial (+)
Habit	Herb (+)	Herb (+)
Tall	70 – 150 cm (+)	25 – 40 cm (-)
Branching	Numerous branches (+)	Numerous branches (+)
Plant not thistle-like	+	+
Plant without latex	+	+
Pappus of bright setae	+	+
Leaf blade	Ovate to elliptic or ovate (+)	Ovate to elliptic or ovate (+)
Leaf dimension	9 – 12 cm×7 – 9 cm (+)	0.2 – 0.3 cm×0.1 – 0.2 cm (-)
Leaf type	3-pinnatisect (+)	3-pinnatisect (+)
Lobules	Lanceolate-elliptic or linear (+)	Lanceolate-elliptic or linear (+)
Lobule dimension	7 – 15 mm×2 – 5 mm (+)	2 – 3 mm×0.5 – 1 mm (-)
Lobule apex	Obtuse apex (+)	Obtuse apex (+)
Leaves alternate	+	+
Adaxial leaf surface	Grayish-green (+)	Dark green (-)
Abaxial leaf surface	Silver-gray (+)	Clear green (-)
Petiole	2 to 6 cm long (+)	Sessile (-)
Stem hair	Pubescence appressed (+)	Pubescence appressed (+)
Stem position	Erect (+)	Erect (+)
Stem color	Greenish gray (+)	Greenish gray (+)
Leafy bracts	3-lobed (+)	Bracts imbricate, multiseriate (-)
Leafy bracts	Entire (+)	Entire (+)
Leafy bracts	Sessile (+)	Sessile (+)
Leafy bracts	Oblong (+)	Oblong (+)
Bracts not spiny-tipped	+	+
Spherical baskets	2.5 – 4 mm in diameter (+)	<3 mm (-)
Florets	2 – 4 in number (+)	2 – 4 in number (+)
Outer tubular flowers	Pistillate (+)	Pistillate (+)
Inner funnel flowers	Bisexual (+)	Bisexual (+)
Flower color	Yellow (+)	Yellow (+)
Corolla	1 – 2 mm (+)	0.5 – 0.75 mm (-)
Corolla	Glandular (+)	Glandular (+)
Capitula	Globose (+)	Oblong (-)
Capitula	3.5 – 4 mm in diameter (+)	0.25 – 0.3 mm (-)
Capitula	Oblong( +)	Oblong (+)
Receptacle	Convex, semi-globose (+)	Convex, semi-globose (+)
Rceptacle	Covered with white ribbon-like scaly films (+)	Naked (-)
Receptacle	Densely hairy (+)	Absent (-)
Involucre 2-seriate	+	+
Heads homogamous	+	+
Cypselas	Oblong (+)	Oblong (+)
Cypselas	Minute upper crown (+)	Minute upper crown (+)
Cypselas	Mostly<0.5 mm (+)	Mostly<0.5 mm (+)
Cypselas	Glabrous (+)	Glabrous (+)

**Table 11:** Root and stem anatomical characteristics of the two plant species studied

Root and stem anatomical characteristics	*Artemisia absinthium*	*Artemisia herba-alba*
A multilayered exodermis in root	+	+
Below the exodermis, a cortex is present in the root	+	+
Below cortex, secondary phloem and some groups of sclerenchyma fibers in root	+	+
The secondary xylem is the dominant part of the root composed of vessels and tracheids	+	+
Endodermal secretory canals are present in the roots	+	-
Root secretory canals, endodermal	+	-
Root secretory canals, nonendodermal cortex	+	-
Root secretory canals, secondary phloem	-	-
Irregular pentagonal shape in the young stem	+	+
More or less round or polygonal in old stem	+	+
One-layered epidermis, composed of oval to isodiametric cells of stem	+	+
Prominent ribs in young stems contained collenchyma ribs in young stems contained collenchyma tissue	+	+
Chlorenchyma is present between the ribs in the stem	+	+
The periderm is continuous in older stems and consists of several layers of enlarged cells arranged in radial rows	+	+
The vascular bundles are collateral and arranged in a circle. The vascular bundles are collateral and arranged in a circle separated from one another by a parenchyma tissue in stem	+	+
The primary xylem consists of four to eight parallel rows of xylem elements; each row comprises 2 – 5 vessels. The vascular cylinder in a secondary state of growth produces secondary xylem inside and a secondary phloem outside, giving more basal parts of the stem an almost cylindrical outline, while medullary rays on the xylem side form connective tissue of lignified cells in stem	+	+
Well-lignified sclerenchyma is above the phloem in the stem	+	+
A large parenchyma cells are in the central region of the stem	+	+
Small secretory canals in the cortex of the stem	+	+
Small secretory canals in the pith in the stem	+	-
Numerous non-glandular as well as glandular trichomes on stems	+	+
The non-glandular trichomes are T-shaped, with various variable numbers of cells that form a neck of the trichome, and with long curly or straight arms in the stem surface	+	+
Glandular trichomes are of biseriate type covered with cuticle sheath in the stem	+	+
Stem secretory canals, cortex	+	+
Stem secretory canals, pith	+	-

**Table 12:** Leaf anatomical characteristics of the two plant species studied

Leaf anatomical characteristics	*Artemisia absinthium*	*Artemisia herba-alba*
Leaf shape on cross-section, oblong-linear	+	+
Leaf secretory canals, phloem	-	+
Leaf parenchyma sheath surrounding vascular bundle extends to both epidermises	+	-
Leaf sub-epidermal collenchyma	+	-
Leaf on the cross-section is oblong-linear	+	+
Palisade tissue, on both leaf sides, consists of large rich in chloroplast cells, arranged in one or two layers.	+	+
In the central leaf blade plane one large collateral closed vascular bundle	+	+
Main vein is prominent with two lateral ribs, which are with many valleculae.	+	+
The vascular bundle in the main vein of leaf is surrounded by a parenchyma sheath extending to both epidermises.	+	-
Secretory canals of the leaf parenchyma	-	+
Numerous non-glandular as well as glandular trichomes on leaves	+	+
The non-glandular trichomes are T-shaped, with various variable numbers of cells which form a neck of the trichome, and with long curly or straight arms in leaf	+	+
Glandular trichomes are of biseriate type covered with cuticle sheath in leaf	+	+
Surface AB shape irregular	+	-
Surface AB shape elongated	+	+
Surface AD shape irregular	+	-
Surface AD shape elongated	+	+
Surface AB margin smooth	+	+
Surface AD margin smooth	+	+
Surface AB length <30 μm	-	+
Surface AD length <30 μm	-	+
AB anomocytic stomata	+	+
AD anomocytic stomata	+	+
AB stomata length >20 μm	+	+
AD stomata length >20 μm	+	+
AB stomata width >15 μm	+	+
AD stomata width >15 μm	+	+
AB guard cell length >15 μm	+	+
AD guard cell length >15 μm	+	+
AB guard cell width >7 μm	+	+
AD guard cell width >7 μm	+	+
Stomata aperture AB length >13 μm	+	-
Stomata aperture AD length >13 μm	+	-
Stomata aperture AB width >4 μm	+	+
Stomata aperture AD width >4 μm	+	+
No. of subsidiary cells on AB surfac e<5	-	+
No. of subsidiary cells on AD surface <5	-	+
Stomata complex AB length <35 μm	-	+
Stomata complex AD length <50 μm	-	+
Stomata complex AB width <40 μm	-	+
Stomata complex AD width <50 μm	-	+

Abbreviations: AB: Abaxial; AD: Adaxial.

**Table 13:** Palynological characteristics of the two plant species studied

Palynological characteristics	*Artemisia absinthium*	*Artemisia herba-alba*
Polar axis	20.47 μm (+)	19.02 μm (-)
Equatorial axis	16.62 μm (+)	18.14 μm (-)
P/E sphericity	1.23 (+)	1.05 μm (-)
Exine thickness	3.03 μm (+)	2.42 μm (-)
Aperture length	12.58 μm (+)	11.67 μm (-)
Msocolpium	3 μm (+)	2.5 μm (-)
Apocolpium index	1.5 μm (+)	1 μm (-)
Shape classes	Peroblate (+)	Peroblate (+)
Size classes	Small grain (+)	Small grain (+)
Amb shape	Elliptic (+)	Elliptic (+)
Pollenkitt	Conspicuous (+)	Non-conspicuous (-)
Aperture width	0.5 μm (+)	0.83 μm (-)
Aperture type	Colpus (+)	Colpus (+)
Aperture number	Tri-aperture (+)	Tri-aperture (+)
Aperture site	Circumaperture (+)	Circumaperture (+)
Aperture membrane	Absent (-)	Present (+)
Aperture surface level	Opening slit (+)	Opening furrow(-)
Exine sculpture	Clavate (+)	Echinae (-)
Polarity	Isopolar (+)	Isopolar (+)
Symmetry	Bilateral symmetric (+)	Bilateral symmetric (+)
Polar area index	0.09 (+)	0.04 (-)
Spinule height	0.48±0.084 μm (+)	0.2±0.042 μm (-)
Spinule spacing	0.96±0.27 μm (+)	0.68±0.12 μm (-)
Spinule ratio	0.5 (+)	0.29 (-)
Sculptural density	2 spinule/μm^2^ (+)	3 spinule/μm^2^ (-)

**Table 14:** Similarity percentage of the two plant species studied

Plant	Characters	Similarity (%)
Morphology	Habitat	85.71
	Leaf	77.78
	Stem	100
	Petiole	0
	Bract	80
	Inflorescence	61.54
	Cypselas	100
Anatomy	Leaf	61.54
	Stem	88.24
	Root	57.14
	Leaf epidermis	50
	Stomata	60
Palynology	Qualitative (12)	66.67
	Quantitative (13)	0

### 3.3. Statistical analysis

**Figure 13:**
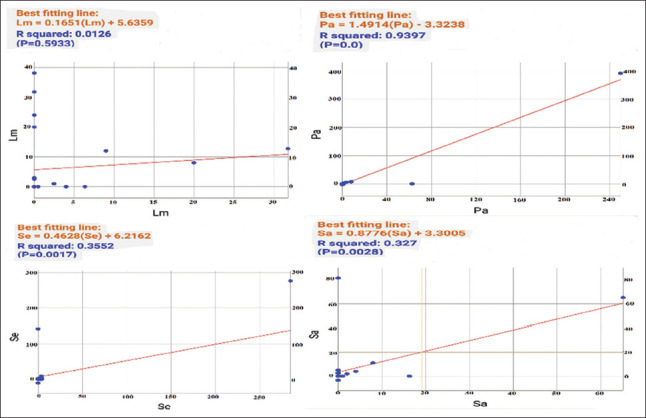
Simple linear regression curves of minimum inhibitory concentrations of both plant species extracts against the tested bacterial strains Abbreviations: Lm: *Listeria monocytogenes*; Pa: *Pseudomonas aeruginosa*; Sa: *Staphylococcus aureus*; Se: *Salmonella enterica*.

**Figure 14:**
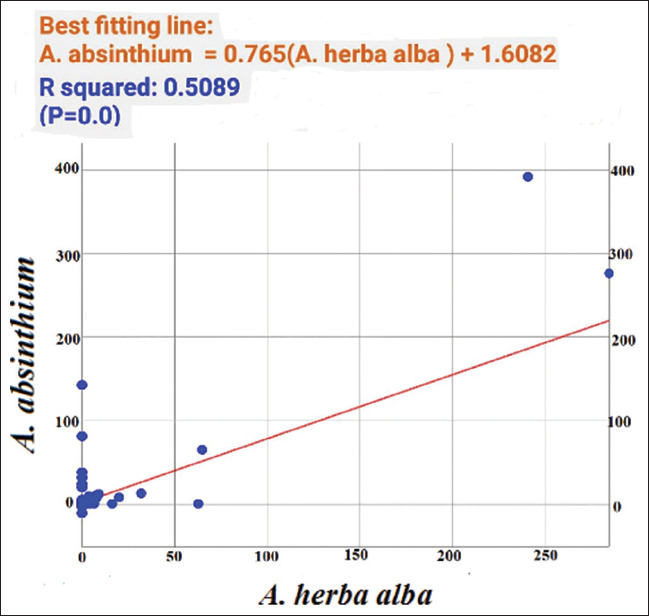
Simple linear regression curve for all comparative data of both plant species

The ANOVA for various MIC values using ANOVA showed significant differences for all tested bacterial strains. In *A. absinthium* extracts, *L. monocytogenes* versus *P. aeruginosa* showed high significance with an *F* value of 51.01. In contrast, *P. aeruginosa* versus *S. enterica* exhibited low significance with an *F* value of 1.64. In *A. herba alba* extracts, *L. monocytogenes* versus *S. enterica* yielded high significance with an *F* value of 111.61 while *P. aeruginosa* versus *S. enterica* presented low significance with an *F* value of 2.57. Pearson correlation coefficients among MICs of *A. absinthium* and *A. herba alba* extracts against the tested bacterial strains demonstrated that *P. aeruginosa* had a highly positive correlation. In contrast, *S. enterica* and *S. aureus* had moderate correlations and *L. monocytogenes* had a low correlation. Regression analysis described the co-variation among MIC variables. The SLR curve indicated significant relationships between them with extremely high regression in *P. aeruginosa*, high regression in both *S. enterica* and *S. aureus* and moderate regression in *L. monocytogenes*. Finally, all comparative data between the two studied plant species differentiating into antibacterial characteristics and morphological, anatomical, and palynological traits were represented by an SLR curve, showing high regression (Table 15, Figures 13 and 14).

**Table 15:** Pearson correlation coefficients among minimum inhibitory concentrations of both plant species extracts against the tested bacterial strains

	*Listeria monocytogenes*	*Pseudomonas aeruginosa*	*Salmonella enterica*	*Staphylococcus aureus*
*Listeria monocytogenes*	**0.112**			
*Pseudomonas aeruginosa*		**0.969**		
*Salmonella enterica*			**0.596**	
*Staphylococcus aureus*				**0.571**

## 4. Discussion

The effort to search for medicinal plant extracts exhibiting antimicrobial activity has been gaining momentum since the World Health Organization reports indicated that antimicrobial resistance is on the rise.^34^ The choice of appropriate organic solvent is crucial in the extraction of natural resources, as clearly illustrated in this study. The extraction involves a variety of organic solvents in addition to an aqueous one. Butanol was the most significant extract in this investigation due to its moderate polarity index (4) with low solubility in water (0.43%) compared to other organic solvents. Chloroform ranked as the second-best organic solvent thanks to its lower viscosity (0.57 cP). The third effective extract was diethyl ether, owing to its low polarity index (2.8), low viscosity (0.32 cP), and high solubility in water (6.89%).^35^

In terms of the MICs, *L. monocytogenes* exhibited the highest susceptibility with the lowest MIC value when treated with the butanol extract of *A. absinthium* whereas *P. aeruginosa* demonstrated the greatest resistance, as indicated by a high MIC value. Conversely, when using diethyl ether extract of *A. herba alba, L. monocytogenes* remained the most susceptible with a low MIC value, while *S. enterica* was the most resistant with a high MIC value in both organic solvents investigated for *A. herba alba*. This variation in susceptibility may be attributed to the architecture of cell walls and their interaction with the extracted chemical components, which impact membrane permeability and protein binding potential. Notably, *L. monocytogenes* is classified as Gram-positive, in contrast to other tested bacterial strains, which are Gram-negative.^4^

Due to its low MBCs, *L. monocytogenes* needed a higher inoculum dose than other strains to proliferate in treatments involving *A. absinthium* and *A. herba alba* using chloroform and butanol extracts, respectively. In contrast, *P. aeruginosa* required the lowest inoculum dose across all treatments, suggesting it possesses a higher virulence compared to the other strains.

Maria *et al*.^10^ demonstrated the antibacterial activity of *A. absinthium* using an ethanol extract against *L. monocytogenes* and *S. aureus*. The plant material was sourced from Europe, particularly around the outskirts of Blaj, Alba, Romania. Their findings were corroborated by studies involving additional bacterial strains, such as *P. aeruginosa* and *S. enterica*, also collected from the European regions, notably among Serbian populations. Roman *et al*.^36^ reported a negative inhibitory effect on *S. aureus* when using 40% or 70% ethanol extract of *A. absinthium* while a positive inhibitory effect was observed with 90% ethanol extract. Previous studies indicated that the chemical constituents of *A. absinthium* vary due to factors such as physiological parts,^37^ geographical locations,^38^ the degree of senescence,^39^ and temperature.^40^ This variability implies antimicrobial activities of this plant material vary with different geographical regions.^10^

Studies on micromorphological features, alongside traditional methods, are gaining attention in plant taxonomy.^41^ Additional research on *Artemisia* sp. may either support or contradict current evidence and nuances, though the available data remain inadequate to fully resolve taxonomic issues within the *Artemisia* genus.^42^,^43^ Active components are distributed throughout the plant, not in specific parts of both studied plant species. Therefore, differentiating traits between the two studied species requires additional ultrastructural taxonomic tools, such as pollen grain analysis, additional to traditional morphological methods, to identify the similarities and dissimilarities between them.

This study provided a clear depiction of descriptive data for both plant species and aimed to address the perceptions of taxonomist in the delineation of *Artemisia* species. It paves the way for comparative classification with other *Artemisia* species and aids in the subgeneric classification of the *Artemisia* genus.

*p* values indicated the effectiveness of the two studied plant species for this investigation, reflecting the importance of their natural composition as potential natural alternatives against microbial infections. Pearson correlation coefficients confirmed that both plant species are effective inhibitory agents against *P. aeruginosa*. Furthermore, equations identified the data analysis as exponential curves at the form of ^y=a+bx (^y: the predicted value of y, a: the y-intercept, the value of Y when X is 0, b: is the slope, representing the change in Y for every unit change in X, x is the independent variable on X axis) presented above each curve, revealing compatibility between the two plant species and among bacterial strains.

Based on our research and the aforementioned studies, the use of application of *Artemisia* extracts in new therapeutic protocols against resistant infectious pathogenic diseases is a potential pathway that warrants further considerations.

## 5. Conclusion

The selection of an appropriate organic solvent is crucial for extracting specific plant compounds. Moreover, employing successive plant extraction with different organic solvents provides a comprehensive understanding of the phytochemical profile, and these solvents vary in physical and chemical properties, such as viscosity, polarity and miscibility. In this study, butanol was identified as the most effective solvent due to its broad antibacterial spectrum. Both *A. absinthium* and *A. herba alba* were recognized as potent antimicrobial agents,^44^ making them promising natural alternatives in response to the growing global demand for such solutions. This investigation also highlighted the influence of geographical factors on plant adaptation, as well as the enhancement of micro-botanical traits like pollen grains, which complement traditional taxonomical approaches. Overall, this emphasizes the importance of selecting the right organic solvent when developing natural antibiotic plant extracts, offering valuable insights for pharmaceutical scientists.

## Data Availability

All datasets were achieved, used, and analyzed in the current study. They are included in this manuscript.

## Appendix

**Glossary**

Amb^45^: Outline of pollen grain viewed with one of the poles exactly uppermost.

Aperture^46^: A weak, performed opening of the exine that permits the exit of intra-exinous contents (e.g. pollen tube).

Apocolpium index^45^: Area at a pole, delimited towards the equator by the polar limits of the mesocolpia.

Circumaperturate^47^: Describing a pollen grain with equatorial apertures that are regularly arranged around a circular outline.

Clavate^48^: Spinules of sharp point apex with wide beneath on pollen grain surface.

Colpus^49^: Longitudinal aperture having a length greater than twice its width.

Echinae^48^: Spinules of pointed apex with narrow beneath on pollen grain surface.

Equatorial axis^49^: A line, lying in the equatorial plane, perpendicular to the polar axis and passing through it.

Exine^50^: The main, outer, usually resistant layer of a sporoderm.

Mesocolpium^45^: The area of the grain surface between two adjacent colpi. It is usually delimited transverse lines drawn through the polar ends of the colpi.

P/E sphericity^49^: The ratio of the length of the polar axis (P) to the equatorial axis (E).

Peroblate^49^: Describing the shape of a pollen grain or spore in which the ratio between the polar axis and the equatorial axis is <2.

Polar area index^51^: The ratio of the distance between the apices of two colpi to its equatorial axis.

Polar axis^52^: A straight line connecting the distal and proximal pole of the pollen grain.

Pollenkitt^53^: A sticky material, produced by the tapetum, that may hold pollen grains together during dispersal.

Sculpture^54^: The surface relief, or topography, of a pollen grain.

Sculptural density^55^: The estimated number of sculptural elements in an area of 100 μm^2^ of the surface of the exine.

Spinules^48^: Processes protrude from exine sculpture.
